# Health Benefits of a Variety in Social Activities: Bidirectional Associations with Physical and Cognitive Health

**DOI:** 10.1093/geroni/igaf122.2994

**Published:** 2025-12-31

**Authors:** Sangha Jeon

**Affiliations:** University of Michigan, Ann Arbor, Ann Arbor, Michigan, United States

## Abstract

Engagement in diverse social activities is linked to better cognitive functioning, fewer depressive symptoms, and greater longevity. Social activity variety may provide unique cognitive, emotional, and physical benefits by fostering purpose in life, stimulating cognitive flexibility, and promoting health behaviors, all of which contribute to overall well-being. Yet, most studies rely on cross-sectional or unidirectional models, leaving the temporal relationship between social activity variety and health outcomes unclear. Drawing on longitudinal data from the Health and Retirement Study (2008, 2012, and 2016) with 2,087 participants aged 50 and above, we examined the dynamic interplay between social activity participation and health outcomes, including self-rated health, chronic conditions, and cognitive functioning. Using a Random Intercept Cross-Lagged Panel Model (RI-CLPM), our findings indicate that greater social activity variety predicts improved physical and cognitive health at subsequent time points. Conversely, individuals with better baseline health are more likely to maintain or increase their engagement in diverse social activities over time. These results suggest a reinforcing cycle in which social activity variety and health mutually support one another. Our findings underscore the importance of interventions that foster diverse social participation as a preventive strategy against cognitive and physical decline in older adults.

